# Review of Mechanisms, Pharmacological Management, Psychosocial Implications, and Holistic Treatment of Pain in Fabry Disease

**DOI:** 10.3390/jcm10184168

**Published:** 2021-09-15

**Authors:** Jonathan Niranjan Rajan, Katharine Ireland, Richard Johnson, Karolina M. Stepien

**Affiliations:** 1 Pain Medicine and Anaesthesia Department, Salford Royal NHS Foundation Trust, Stott Lane, Salford M6 8HD, UK; katharine.ireland@btinternet.com; 2 Manchester & Salford Pain Centre, Salford Royal NHS Foundation Trust, Stott Lane, Salford M6 8HD, UK; richard.johnson@srft.nhs.uk; 3 Adult Inherited Metabolic Diseases, Salford Royal NHS Foundation Trust, Stott Lane, Salford M6 8HD, UK; Karolina.Stepien@srft.nhs.uk; 4 Division of Diabetes, Endocrinology & Gastroenterology, University of Manchester, Manchester M13 9PL, UK

**Keywords:** fabry disease, neuropathic pain, depression

## Abstract

Fabry disease is a progressive X-linked lysosomal storage disease caused by a mutation in the *GLA* gene, encoding the lysosomal hydrolase α-galactosidase A. The consequent reduced enzyme activity results in the toxic accumulation of glycosphingolipids, particularly globortriaosylceramide (Gb3 or GL_3_), in blood vessels, renal epithelia, myocardium, peripheral nervous system, cornea and skin. Neuropathic pain is the most common manifestation of Fabry disease and can be extremely debilitating. This often develops during childhood and presents with episodes of burning and sharp pain in the hands and feet, especially during exercise and it is worse with increased heat or fever. It is thought to be due to ischaemic injury and metabolic failure, leading to the disruption of neuronal membranes and small fibre neuropathy, caused by a reduced density of myelinated Aδ and unmyelinated C-fibres and alterations in the function of ion channels, mediated by Gb3 and lyso Gb3. It is important to confirm small fibre neuropathy before any Fabry disease treatment modality is considered. There is a clinical need for novel techniques for assessing small fibre function to improve detection of small fibre neuropathy and expand the role of available therapies. The current Fabry disease guidelines are in favour of pharmacological management as the first-line treatment for pain associated with Fabry disease. Refractory cases would benefit from a rehabilitation approach with interdisciplinary input, including medical, physiotherapy and psychological disciplines and including a Pain Management Programme.

## 1. Introduction

Fabry (sometimes referred to as Anderson–Fabry) disease is a progressive X-linked lysosomal storage disorder caused by a mutation in the *GLA* gene, encoding the lysosomal hydrolase α-galactosidase A (α-Gal A) [1]. This results in the toxic accumulation of glycosphingolipids, particularly globortriaosylceramide (Gb3 or GL_3_), and lyso-Gb3, the decylated form of Gb3 in blood vessels, renal epithelia, myocardium, peripheral nervous system, cornea and skin [1].

The estimated prevalence of Fabry disease is 1:40,000–170,000 [2]. The systemic manifestations of Fabry disease can be divided into early manifestations occurring in childhood and those occurring in a second phase in early adulthood and later life. The implications on organ function are wide ranging and progressive (Table 1).

In untreated males, the median life expectancy is approximately 50 years [6,7]. Mortality is related to cardiac hypertrophy, arrhythmias, cerebral infarction and renal failure if renal replacement therapy is not utilised [1].

Pain is a hallmark of classical Fabry disease and is a significant feature particularly within the first two decades of life. This has implications for normal childhood development, academic progress and family life. Pain has a significant impact on both male and female patients and leads to a significant reduction in quality of life compared to the general population. This appears to coincide with depression, anxiety and chronic fatigue in a significant percentage of people living with Fabry disease [8]. Neuropathic pain and Fabry crisis are typical of the classical phenotype of Fabry in males with no residual enzymatic activity, rather than the non-classical phenotype [9].

This review presents the pathophysiology and investigations of pain in adults with Fabry disease, with a focus on the multidisciplinary team (MDT) management of different types of pain.

### 1.1. Epidemiology of Pain

Acroparesthesiae (neuropathic pain) is the most common manifestation of Fabry disease, occurring in up to 62–80% of classical hemizygous male patients and 30–65.3% of heterozygous female patients [10,11,12]. Long-term observational studies confirmed that a mean onset of pain is 14.8 years for men and 19.8 years for females [12]. The rate of Fabry-specific small fibre neuropathy has been documented in 78% [13] and 50–75% [14] of females affected with this condition.

Although Fabry disease is an X-linked disorder, it is becoming clearer that heterozygote females develop acroparaesthesiae at rates in excess to that seen in the general population [15,16]. However, a significant heterogeneity among individuals with the same mutation in the *GLA* gene makes it difficult to predict which patients will experience pain [17].

Pain in Fabry disease is dependent on the amount of residual enzymatic activity. Hence, pain is a hallmark of disease in the classical phenotype with no residual enzymatic activity. However, there may be a wide spectrum of phenotypes ranging from classical to later onset type in males [18], and a wide variation in pain experienced by heterozygous female [19]. In females this can occur by two mechanisms. The first being lionisation, whereby a copy of the X chromosome is inactivated during the embryonic stage. The second being X chromosome skewing, where the mutant form is expressed to a high degree in certain key tissues. Hence, variability in pain can occur in females with either of these X-linked inheritance patterns [20,21,22].

### 1.2. Types of Pain

Pain often develops during childhood and presents with episodes of burning, sharp, stabbing and shooting pains in the hands and feet, especially during exercise and it is worse with increased heat or fever [23]. Patients often report heat and cold intolerance, intense intermittent pain, hypersensitivity to mechanical stimuli and gastrointestinal pain [17,24].

In females with classic Fabry disease, small nerve fibre dysfunction remains largely restricted to the calf. However, males with classical Fabry disease, show additional involvement of the thigh with disease progression [14,17]. Small fibre neuropathy starts at a distal site at a young age, in both females and males with Fabry disease ascending more proximally only in males with the classical form of the disease at a later stage [14,17].

Evoked pain, triggered by stimuli including brushing, pressure or cold, has been reported in 31–45% of patients [25]. While evoked pain is frequently experienced by Fabry disease patients, pain crises are the most debilitating acute pain experienced by them [17]. Severe episodes of acute limb pain known as ‘Fabry crises’ can last from minutes to weeks and are thought to be caused by small fibre involvement in peripheral nerves. These crises are thought to be triggered by several factors including exercise, changes in ambient temperature, stress, over exertion, concurrent illness, alcohol and heat [23]. These typically occur distally in the hands and feet with pain and acroparesthesia radiating proximally, as the crisis progresses [17]. Pain crises occur weekly to once a year, lasting minutes to weeks [17].

Significant heterogeneity in thermal and mechanical responses among Fabry disease patients depends on severity and progression of the disease.

Additional information is required to determine whether small fibre neuropathy is Fabry disease related or caused by other conditions giving rise to damage to small nerve fibres. Other symptoms and signs of Fabry disease crises include hypohydrosis, reduced cerebrovascular reactivity and labile blood pressure. Post-prandial abdominal pain with alterations in gastrointestinal motility and early satiety have been also described [23]. Many Fabry patients experience severe gastrointestinal pain, both chronic pain between meals and intermittent pain after eating, that is often followed by diarrhoea, bloating, and early satiety [25].

Chronic pain, lasting over 3 months, is reported by 12–40% of patients and affects joints in the legs, shoulders, back, but also abdomen and head [17,26]. It is unclear whether acute pain progresses into chronic pain over time.

## 2. Pathophysiology of Pain

The processes involved in the development of pain in Fabry disease are complex and are thought to be mainly neuropathic in nature, due to the involvement of Lyso-globotriaosylceramide (Gb_3_) on dorsal root ganglia and the peripheral nervous system which lead to cell swelling [27,28].

Gb3 gradually accumulates within the dorsal root ganglia, causing change in its structure, while peripheral nerve morphology is changed to a minimal degree. Gb3 content within dorsal root ganglia neurons has also been documented ex vivo [28] and has been found to be 10-fold higher than in the brain [27]. Abundant Gb3 deposits were documented in perineurial, endothelial and smooth muscle cells but not within peripheral axons potentially relating to an indirect effect of Gb3 deposits on peripheral nerves causing damage due to vasa nervorum obstruction with a subsequent peripheral nerve ischaemia [29]. Gb3 accumulation was documented in all classical Fabry disease patients, with lower skin innervation as compared to Fabry disease patients with late-onset variants or polymorphisms [30].

In one study, exogenous lyso-Gb3 was administered into dorsal root ganglia, and resulted in an increase in cytoplasmic Ca^2+^ levels within the sensory neurons, suggesting a direct effect of lyso-Gb3 on sensory neuron altering nociception and ultimately pain production [31].

In addition, a decreased blood supply in dorsal root ganglia and a hypoxic environment may result in neuronal apoptosis and/or a decreased microvascular supply of the distal nerve segments [32]. The progressive Gb3 accumulation in dorsal root ganglia was also observable both an α-Gal A mouse model and rat model [33,34].

### 2.1. Types of Fibres

Fabry disease neuropathy it is largely thought to be due to ischaemic injury and metabolic failure, leading to the disruption of neuronal membranes and small fibre neuropathy caused by a reduced density of myelinated Aδ and unmyelinated C-fibres [35,36]. This leads to impaired temperature and pain transmission. The remarkable small fibre loss is most pronounced in the distal long axons of the lower extremities. The fibre loss has been found to be substantially higher in the skin than in the peripheral nerve trunk [37].

While there is minimal impact on nerve conduction in large-diameter, myelinated Aβ-type fibres, there is impaired function and loss of cutaneous Aδ- and C-sensory neurons [26,38].

Cold sensation can be transmitted through polymodal C-fibres and Aδ thinly myelinated fibres, and harmful heat and gentle touch is transduced though mechanically sensitive C-fibres [39]. Therefore, small fibre neuropathy results in hyposensitivity to warmth, cold and touch. The presence of Fabry-specific small fibre neuropathy features should be obligatory to confirm diagnosis and start therapeutic interventions.

### 2.2. Ion Channels

Several ion channels have been associated with pain including, voltage-gated sodium, potassium, and calcium channels, transient receptor potential (TRP), acid sensing (ASIC), and hyperpolarisation-activated cyclic nucleotide-gated (HCN) ion channels. In Fabry disease, the slowing of action potential generation from nerve fibres, which is commonly related to sodium channel dysfunction, was not affected, indicating that sodium channel function and expression is normal in C fibres from Fabry disease patients [39,40]. In another study, potassium channel abnormalities and increased sensory neuron depolarisation correlated with pain severity [41].

Further changes in voltage-dependent sodium channel (including Nav1.3, Nav1.8, and Nav1.9) [33,39], voltage-gated potassium channels (including KCNB2) [39,42], voltage-gated calcium channels (including CACNA1H) [31,42], and transient receptor potential (TRP) channels in Fabry rodents (including TRPV1 and TRPA1) [33,42] have been observed in animal models that displayed pain behaviour [43].

### 2.3. Reduced Perfusion

The characteristic pathophysiological vasculopathy seen in Fabry patients causing an alteration of nitric oxide liberation, leading to an abnormal vascular response and reduced perfusion, could be linked to pain conditions associated with physical stress in Fabry. Politei et al. (2016) suggested that the application of capsaicin cream or lidocaine may reduce physical stress induced pain in Fabry patients [44]. In particular, capsaicin leads to a distinct increase in blood perfusion and thereby could attenuate these specific pain conditions [36,45]. It has also been reported that p75 neurotrophin receptor (p75^NTR^) plays a critical role in nerve growth factor (NGF)-induced sensitisation of capsaicin-sensitive small-diameter sensory neurons [46,47] and that the majority of capsaicin-responsive neurons respond to globotriasylsphingosine, a deacylated form of Gb3 [31].

### 2.4. Inflammation

Inflammation has been postulated as a potential pathomechanisms of Fabry disease [48] with NGF being the key molecule mediating pain [49,50,51,52]. In experimental studies, treatment with a neutralising antibody against a precursor of NGF (proNGF) or its receptor, p75^NTR^, led to the improvement in Gb3-induced allodynia [52]. It has been suggested that toxic concentration of Gb3 activates the pain pathway mediated through functional upregulation of proNGF–p75^NTR^ signalling [52].

In addition, in vivo studies have shown involvement of pro-inflammatory cytokine mediators in the immune system, including TNF-alpha, IL-1beta, TLR4, and IL-6, suggesting that pain, apart from being neuropathic, may also encompass inflammatory components [53,54].

### 2.5. Oxidative Stress

Lysosomes degrade and recycle organelles in a process known as autophagy. The degradation of mitochondria is known as mitophagy. Mitophagy prevents the build-up of reactive oxygen species (ROS) in cells including neurons [25]. The effects of ROS may lead to a reduction in the ability to produce ATP in cells which have a limited scope to increase glycolytic production of ATP. As such, it may not then be possible for the ATP-dependent proton pump to maintain acidity in the lumen of lysosomes, which may have a role to play in neuronal dysfunction and pain in FD [55]. GB3 has been shown to increase the production of ROS in Fabry disease patients. Since this effect is maintained in the plasma of Fabry disease patients despite reductions in intracellular GB3 other factors in the plasma may be contributing to the production of ROS [56].

The effect of GB3 on inflammation and ROS is complex. The intracellular accumulation of GB3 leads to a reauction in eNOS and a concomitant increase in the iNOS, leading to NO production and increased ROS. Furthermore, ROS reduced glutathione (GSH) levels. GSH is the key non-enzymatic antioxidant, which preferentially oxidises key reactive species thus preserving more important biomolecules [57,58].

ROS lead to an alteration in superoxide dismustase:catalase activity ratio (SOD), which is a key antioxidant within mitochondria [55]. Its downregulation may allow for greater ROS effects on the mitochondrial respiratory chain. (MRC) Whilst evidence for the contribution of ROS comes mainly form studies in cardiac cells, vascular endothelium and renal disease, it raises the question as to whether such effects in neuronal cells, are integral to pain in Fabry disease [59].

### 2.6. Additional Mechanisms

There is some evidence that cholinergic nerve fibre dysfunction contributes to the altered nitric oxide metabolism in Fabry disease [36]. Moreover, a dysfunctional cholinergic response may explain the characteristic general heat intolerance observed in Fabry patients [60], due to its co-regulation in response to whole-body heating [61].

Central sensitisation and altered pain modulation are also likely involved in patients with Fabry disease [44]. Intermittent peripheral neuropathic pain in individuals sensitive to multiple stress factors may sensitise the central neural pain system, resulting in amplification of pain in the body. It has been postulated that central sensitisation of pain may also contribute to fatigue, sleep disorders and mood fluctuations in patients with Fabry disease [44].

Pain in Fabry disease also consists of nociceptive or inflammatory components, as demonstrated by its responsiveness to non-steroidal anti-inflammatory drugs (NSAIDs), in many Fabry disease patients [15,16,17]. It has been postulated that some of the spontaneous types of pain in patients with Fabry disease may be associated with hyperexcitability of peripheral nociceptive neurons affected by upregulation of sodium ion channels (Nav1.8) [33], TRPV1 [45], or an increase in calcium (Ca^2+^) influx which is lyso-Gb3 dependent [31].

## 3. Investigation of Pain

In heterozygote females, in the absence of other manifestations of Fabry disease, confirmation of small fibre neuropathy using insensitive traditional techniques (e.g., quantitative sensory testing, quantitative sudomotor axon reflex test, skin biopsy and assessment of intraepidermal nerve fibre density) is usually required before commencement of enzyme replacement therapy (ERT) or any other treatment modality is considered [44].

The severity of neuropathy closely correlates with plasma levels of Gb3 or its metabolites [35,44]. Unfortunately, small nerve fibre function (A-δ and C-fibres) is difficult to accurately quantify using current techniques (e.g., quantitative sensory testing) as high levels of patient cooperation are required, making it inherently difficult to minimise subjectivity.

The advantage of skin biopsy is its effectiveness in assessing small fibre neuropathy when compared to electromyography and nerve conduction studies. It can be also repeated multiple times to monitor disease progression or treatment efficacy [62].

In such cases, however, it is also likely that improvements in response to ERT or chaperone therapy will be difficult to detect given the level of structural nerve fibre damage that has already occurred.

There is a clinical need for novel techniques for assessing small fibre function to improve detection of small fibre neuropathy and expand the role for the available therapies [35] (Table 2).

Microneurography is a minimally invasive technique which involves using a microelectrode (fine electrical recording needle) to record nerve activity in a superficial nerve [63,64]. The method is technically demanding and lasts many hours hence it is not suitable as a routine diagnostic tool to diagnose small fibre neuropathy. A key advantage of microneurography is the ability to detect different profiles of neural activity corresponding to different subpopulations of sensory neurons and peripheral nociceptors. Microneurography has only been previously performed in some studies of patients with Fabry disease heterozygote females [49]. By detecting evidence of small fibre dysfunction (i.e., hyperexcitability of C-nociceptors) prior to the onset of significant structural nerve damage, microneurography has the potential to improve the ability to detect small fibre neuropathy in patients with Fabry disease and in manifesting heterozygote females. As has been demonstrated for renal and heart disease in Fabry disease, it is thought that improved detection, and hence early treatment, of small fibre neuropathy in patients with Fabry disease and heterozygote females will be associated with improved clinical outcomes, reducing or delaying permanent nerve damage [65].

## 4. Available Therapies for Fabry Disease

The recent development of enzyme replacement therapy (ERT) for Fabry disease has revolutionised outcomes and has become the standard of care in affected patients [66]. Other options include pharmacological chaperone therapy (Migalastat) [67] as well as novel emerging therapies including Lucerastat [67] and gene therapy [68,69].

ERT has been shown to not only prevent major organ complications, but also to reduce neuropathic pain. Although ERT did not impact intra-epidermal nerve fibre density [15], small nerve fibre function improved as a result of ERT [12,70], with differences from baseline detected after 18 months treatment [12].

Whilst pain-related quality of life has been shown to improve in response to ERT, a significant proportion of patients respond incompletely, presumably because of the presence of irreversible nerve damage [71]. In children, ERT has also been shown to have a positive effect on pain [72].

Several studies investigated treatment response to ERT. In one placebo-controlled trial, Gb3 clearance in skin from the back was observed 20 weeks after the initiation of ERT [73]. Another study assessed skin Gb3 under ERT and showed its partial clearance after 3 years of the therapy [74]. However, the study by Uceyler et al. (2014) did not observe any difference in the Gb3 load of patients on ERT compared to treatment naïve group [75].

Migalastat is an oral pharmacological chaperon therapy (daily tablet), which stabilises amenable mutant forms of α-Gal A in the endoplasmic reticulum, thus facilitating passage to the lysosomes and enzymatic breakdown of Gb-3. The pain severity component of the Brief Pain Inventory (BPI) showed that patients had mild pain at baseline and it remained stable over the 18 month treatment period, when patients were treated with Migalastat [76]. Whilst Migalastat shows promise in cardiac outcomes in patients with Fabry disease, its effect on pain and quality of life are still to be determined.

Lucerastat, an iminosugar, is currently under investigation in a phase 3 study of Fabry disease with neuropathic pain as the primary end point (MODIFY study: CLinicalTrials.gov Identifier: NCT03425539).

Gene therapy is the delivery of a therapeutic gene for cellular expression to alter a disease phenotype. Recombinant lentivirus base gene therapy and adeno-associated virus therapy have begun in Fabry disease [69]. Gene editing including repairing disease-related mutant genes may also have a role in slowing the disease progression in Fabry patients in future [69]. Trials on gene therapy in Fabry disease are open for recruitment around the world (ClinicalTrials.gov Identifier: NCT04519749; NCT04046224; NCT04040049; NCT04455230; NCT03454893).

## 5. Analgesic Agents

### Treatment Goals in Fabry Disease

Pain therapy in Fabry disease remains an unmet need. When considering therapeutic goals with respect to pain in Fabry, patients can be divided into two groups, namely, patients with pain and significant associated distress, and those with predominantly neuropathic pain without overt distress. The priority in the first group is to optimise their analgesics and implement holistic support, whilst in the second group a largely medical approach is employed. Patients should be encouraged to adopt pain diaries and the BPI and SF36 (Short form 36) or EQ5D uses as quality of life scores on a 6 monthly basis [44,77].

Despite clarity with respect to therapeutic goals, there appears to be a limited consensus on how best to effectively manage pain in this cohort of Fabry disease patients. Further to recent European recommendations on the pain management in Fabry disease [43], a systematic review by Schuller et.al (2016) analysed 731 articles and found a range of different therapies being offered with no clear guidance [78]. Amalgamating recent expert guidance and our own experience we present an analgesic ladder suitable for Fabry disease [44,77] (Table 3).

Gastrointestinal symptoms and abdominal discomfort can be managed with dietetic recommendations regarding meal content, portion sizes and frequency. Nausea and vomiting can be treated with metoclopramide and domperidone, with motilin receptor agonists added in case of severe symptoms [44]. Frequent bowel motions can be managed with loperamide. There is increasing evidence from the phase 3 FACETS trial that Migalastat reduces diarrhoea in patients with Fabry disease [83,84].

A multidisciplinary approach is important when forming an analgesic care plan involving not just metabolic and pain specialists, but also specialist pharmacists, pain psychologists and physiotherapists in the service. The management of pain in Fabry disease is challenging as a result of the unique interplay between a number of factors. Neuropathic pain, which is normally challenging to treat, is combined with a presentation in patients with multiorgan dysfunction, often limiting pharmacological management. In addition, the most severe symptoms are often found in males starting in their youth at a time of great flux in their psychosocial development. Fabry disease may be well recognised within the family unit but poorly recognised and understood by the medical community as a whole.

## 6. Psychology Support in Pain Management

### 6.1. Mental Health and Pain in Fabry Disease

High rates of depression have been reported in patients with Fabry disease. Pain is a significant contributory factor to depression [85] and anxiety [8,23]. However, biopsychosocial factors may also contribute to the mood disorder in Fabry disease [86]. Overall, it is estimated that Fabry patients experience higher rates of depression (27–57% of patients) compared to the general population (7–27%) [23,85]. A recent study found that as many as 46% of patients have depression and 28% could be classified as having severe clinical depression [87].

Given the range of developmental, physical, and neurological challenges associated with Fabry disease, its association with depression is unsurprising. However, faced with this context, both patients and clinicians are at risk of viewing such negative psychological associations as an inevitable consequence of Fabry disease. Indeed, this perception is consistent with the finding that the magnitude of decrement in social adaptive functioning correlates with anxiety and depression [60]. However, Laney et al. (2010) also note that in their study, decreased social adaptive functioning was not significantly associated with disease severity, pain, or level of vitality [88]. This finding implies that other variables are involved in the mediation of Fabry disease and negative social, functional, and psychological outcomes. Candidates include modifiable factors such as coping behaviours which can therefore form treatment targets of psychological and rehabilitative approaches, as in other pain conditions, such as chronic low back pain [89].

In Fabry disease, examples of such modifiable factors include behavioural and psychological moderators (such as overexertion and stress) of physical symptoms such as pain. The cognitive model underpinning cognitive behavioural therapy [90] emphasises the role of cognitions (e.g., appraisals, perceptions) in shaping behavioural, emotional and concomitant physiological responses. Psychological intervention with such moderators of symptoms in Fabry disease may therefore involve helping patients to identify and manage environmental, cognitive and emotional factors which can drive unhelpful behaviours such as overexertion; or unhelpful physiological states such as stress.

Psychological treatment in the form of cognitive behavioural therapy has been used in the treatment of anxiety and depression for several decades [91,92], and continues to be a mainstay of treatment presently [93,94]. Similarly, the approach has been shown to be effective in improving psychological status, function, and health outcomes in a range of physical health conditions [95]. Similarly, a recent study, Ali et al. (2018) demonstrated the utility of psychological treatment in improving depression and quality of life in participants with Fabry disease [25].

Depressive symptoms have a significant impact on neuropsychological functioning in up to 16% of patients [96]. Therefore, there is a clinical need for future psychological treatment tailored to coping styles.

### 6.2. Pain Management Programme

The best evidenced rehabilitation treatment for patients with persistent pain and disability is the group Pain Management Programme. These programmes are an interdisciplinary treatment approach including medical, physiotherapy and psychological input and are based on Cognitive Behavioural Therapy, typically providing around 36 h of rehabilitation treatment [97].

Whilst the majority of patients attending Pain Management Programmes have musculoskeletal pain, the British Pain Society (2013) notes that “a minority have visceral, neuropathic, phantom or central pain, and/or pain from identified disease such as osteoarthritis and rheumatoid arthritis”. Therefore, Pain Management Programmes are effectively used in our tertiary Adult Metabolic Centre for patients with Fabry disease.

### 6.3. MDT in a Tertiary Metabolic Centre

Our model of working with Fabry disease (along with other metabolic conditions) has evolved to encompass joint metabolic and pain clinics, involving both patients and their carers. We see on average 4–5 Fabry patients per joint clinic, the majority of whom are male and with moderate to high psychometric scores. This allows a combination of expertise in metabolic and pain medicine and the involvement of a selected number of the rehabilitative interdisciplinary team, featuring psychologists, pain physiotherapists and occupational therapists.

At our institution, pain in Fabry disease patients is managed in a holistic manner. Adult and transitional patients with pain first have access to the pain clinic in a joint clinic often with parents and carers. As part of this assessment, routine care with involvement of other specialties is initiated and a range of psychometric scores are completed by the patient, to act as a baseline and help inform the next steps in holistic control and management of pain and quality of life. These psychometric scores include, the PHQ-9 score, GAD-7 score, TSK and PCS scores, to assess for depression, anxiety, kinsesiophobia and catastrophisation, respectively. Specific Fabry questionnaires such as the Fabry scan questionnaire may also be employed [25]. This is a 15-item questionnaire with bedside sensory function tests of both small and large fibres. It may help distinguish Fabry disease from other chronic neuropathic pain. Alternatives include the Fabry-specific paediatric pain questionnaire and the Würzburg Fabry pain questionnaire which consists of 22 open questions addressing the four main types of pain in Fabry disease [44]. More generic neuropathic pain questionnaires, such as the Pain DETECT questionnaire may also help establish a baseline but are not validated for Fabry disease.

Physiotherapy and psychology colleagues work collaboratively to address issues such as pacing, exercise and activity medication, passive cooling strategies, sleep hygiene, goal setting, flare up management, confidence, and identity. Pharmaceutical agents are used alongside this where appropriate.

The interdisciplinary team must be mindful of the impact of Fabry disease on educational attainment, marital status, social support and employment which are significantly affected by the effects of the disease [6].

As an example, a 45-year-old female patient with Fabry disease benefited from this treatment at our institution. The preliminary assessment identified that stressors often aggravated her pain symptoms, and this in turn led to avoidance of potentially stressful situations, which then contributed to increased disability. Activity cycling was also a feature. This pattern involves determined efforts to undertake activities, which fuels overexertion and is then followed by an increase in pain and reduction in activities. Treatment goals developed with the Pain Management Programme Team included (i) increasing understanding and effective management of anxiety to reduce the impact of stress on pain, (ii) improved stress management skills to enable a reduction in avoidant coping and thereby to reduce disability, (iii) problem-solving skills to manage work demands and avoid activity cycling, and (iv) improved activity pacing skills to enable a return to leisure activities. Standard physical outcome measures showed significant gains in physical function after the Pain Management Programme. Psychometric outcome measures at 12 months post-programme showed substantial improvements in depression, anxiety, and pain catastrophising.

## 7. Avoiding Precipitating Factors

In addition it is recommended that activities that may trigger painful crises such as emotional stress, physical exertion and temperature changes are minimised [77].

Patients are advised to monitor when painful crises occur and to try to avoid anything which may precipitate a crisis. Passive cooling strategies can be utilised to improve tolerance to exercise and heat induced pain. Gastrointestinal pain can be managed with smaller meals, low-fat diets and motility agents whilst the use of antianginal agents may also be helpful for ischaemia angina pectoris-related pain.

## 8. Conclusions

The management of pain in Fabry disease is challenging as a result of the unique interplay between a number of factors. Pain in Fabry disease may affect patients from childhood. Novel techniques in detecting small fibre neuropathy are needed, whilst traditional disease modifying treatments have a limited impact on pain.

Neuropathic pain, which is not normally easy to treat, is further complicated by patients with multiorgan dysfunction, often limiting pharmacological management. In addition, the most severe symptoms are often found in males starting in their youth, at a time of great flux in their psychosocial development.

Ultimately, patients may derive great benefit from interdisciplinary rehabilitation. A multimodal pharmacological approach using a combination of neuropathic agents such as the gabapentinoids and duloxetine may facilitate a reduction in neuropathic pain whilst achieving an opioid-sparing effect. In turn, a reduction in neuropathic pain may also facilitate greater engagement with interdisciplinary rehabilitation to achieve a better quality of life.

## Figures and Tables

**Table 1:** Systemic manifestation of Fabry disease at early and advanced staged of the disease. Early phase systemic manifestations, excluding bradycardia, affect only those with classical Fabry disease [3].

Phase	System Involved	Manifestations
Early Phase	Neurological	Acroparesthesia, Neuropathic-type pain
	Gastrointestinal	Irritable bowel disease like symptoms; diarrhoea, abdominal pain, cramps nausea, vomiting
	Psychological	Behavioural issues Anxiety Depression Vascular dementia
	Ophthalmic	Corneal opacities, corneal verticillata
	Cardiovascular	Bradycardia–T wave inversion increased increased PR interval (other cardiovascular complications unusual in early phase) [2,4,5]
	Autonomic	Hyporhidrosis
	Dermatological	Angiokeratomas corporis diffusum Lymphedema
	Ear Nose and Throat	Impaired hearing
Second Phase	Nephrological	Proteinuria and microalbuminuria
	Reproductive	Azoospermia
	Musculoskeletal	Reduced bone mineral density and disuse atrophy Secondary mitochondrial changes
Late Phase	Cardiac	Diastolic and systolic dysfunction, arrhythmias including atrial fibrillation, atrial flutter and sinus node disease Ischaemia, myocardial fibrosis, left ventricular hypertrophy valvular regurgitation Sudden cardiac death
	Cerebrovascular	White and grey matter changes Transient ischaemic attacks, strokes Vascular dementia

The PR is the interval between the start of the P wave and the start of the QRS complex. A normal interval is between 0.12 s to 0.2 s.

**Table 2:** Summary Systematic Guide to diagnosing and investigating the pain in Fabry disease [44].

Investigation of Pain	Diagnostic Cues and Tools
History	Burning pain in hands and feet (‘glove and socks’ distribution) Pain preventing sporting activities Symmetrical distribution Pain gets worse in high or low temperatures Family history of similar complaints History of pain crisis Pain does not respond to analgesic agents
Differentials	Diabetic polyneuropathy Post-herpetic neuralgia, radicular pain Autoimmune neuropathy Amyloidosis Complex regional pain syndrome Eryromelagia Idiopathic small fibre neuropathy
Neuropathic Pain Screening tools	Neuropathic pain symptom inventory (NPSI) Neuropathic pain questionnaire (NPQ) Doleur neuropathique 4 (DN4) Pain DETECT Leeds assessment of neuropathic signs and symptoms questionnaire (LANNS)
Screening specific to pain	Fabry-specific paediatric health questionnaire (paediatric patients only) Fabry scan questionnaire Wurzburg Fabry pain questionnaire (pain history, character, triggers, impact on quality of life) BPI SF36
Somatosensory Testing Specialist Tests	Quantitative sensory testing Nociceptive evoked potentials Skin biopsy for intraepidermal nerve fibre density nerve conduction studies (to assess for associated carpal tunnel syndrome)
Somatosensory Testing bedside tests	Thermal perception tests Cold perception tests Tests of sensation to pinprick and soft touch Vibration testing

**Table 3:** Analgesic ladder for the treatment of Fabry disease [77,79,80,81,82].

Agent	Mechanism of Action	Dose	Side Effects	Cardiac Caveats	Renal Caveats
First Line					
Tricyclic antidepressant - Amitriptyline - Nortriptyline	5HT and NA reuptake Inhibition. Action on dopaminergic pathways and locus coeruleus.	12.5–150 mg/day	Dry mouth, sedation, arrythmias, urinary retention Diarrhoea, cognitive disturbance, worsening of autonomic instability		Reduce dose in renal impairment
Serotonin and noradrenaline reuptake inhibitors - Duloxetine - Venlafaxine	5HT and NA reuptake Inhibition.	60–120 mg/day 150–225 mg/day	Serotonergic syndrome Gastrointestinal discomfort diarrhoea, anxiety, dizziness	Caution: arrhythmogenic Monitor QTc interval	Reduced dose if eGFR < 30 Reduce dose in renal impairment
Carbamazepine	Reduced Na + channel conductance. Reduction in ectopic discharges.	250–800 mg/day in two divided doses	Associated with blood dyscrasias, Steven’s Johnson’s syndrome, toxic epidermal necrolysis and hyponatraemia		None
Gabapentin	Inhibit calcium mediated neurotransmitter release through effects on *α*_2_ *δ*-1 subunits. NMDA receptor antagonism.	Titrated from 100 mg/day to 3600 mg/day in three divided doses	Weight gain, cognitive dysfunction, lethologica		Reduce dose in graduated fashion with renal impairment
Pregabalin	As Gabapentin.	Starting dose 50 mg bd up to 300 mg bd	As Gabapentin		Reduce dose in renal impairment
Second Line					
Intravenous lidocaine	Local anaesthetic causing sodium channel blockade.	2–5 mg/kg			None
Topical capsaicin	Depletion in substance P.	0.0125% applied topically for 12 h daily	Burning, pruritus		None
Tramadol	Noradrenaline and serotonin reuptake inhibitor. Mu (µ) opioid receptor agonist.	100–400 mg/day	May lower seizure threshold		Caution in renal insufficiency
Third Line					
Strong opioids Morphine oxycodone	Opioid receptor agonists.	30–120 mg 12 hourly 20–60 mg 12 hourly	Nausea, constipation, itch, respiratory depression, osteoporosis, reduced immunity, endocrine dysfunction		Caution in renal insufficiency
Cannabinoids	Stimulation of CB1 and CB2 receptors, action on serotoninergic receptors.		Decreased appetite, nausea, vomiting, fatigue, mood changes, suicidal ideation		
Fourth Line					
Methadone	NMDA antagonist activity Noradrenaline reuptake inhibition and mu-opioid receptor agonist.	50 mg BD maximum 500 mg daily	As per strong opioids	Risk of QTc prolongation	
Tapentadol	Sodium channel blockade and supressed release of glutamate.	25 mg once daily for 2 weeks up to 400 mg daily.	Anxiety; anorexia asthenia; diarrhoea; heat or cold intolerance, gastrointestinal discomfort; muscle spasms; sleep disorders; tremor	May cause Brugada syndrome	
Less efficacious anti-convulsants (Lamotrigine)			Aggression; agitation; arthralgia; diarrhoea; dizziness; drowsiness; dry mouth; fatigue; headache; irritability; nausea; pain; rash; sleep disorders; tremor; vomiting		

## Data Availability

Data available on request due to restrictions e.g., privacy or ethical. The data presented in this study are available on request from the corresponding author. The data are not publicly available due to protecting the privacy of subjects.

## Abbreviations

ASIC	Acid-Sensing Ion Channels
BPI	Brief Pain Inventory
ERT	Enzyme Replacement Therapy
GAD-7	Generalised Anxiety Disorder-7
HCN	Hyperpolarisation-Activated Cyclic Nucleotide-Gated
Lyso-Gb3/Lyso-GL3	Globotriaosylsphingosine
NGF	Nerve Growth Factor
NSAIDs	Non-Steroidal Anti-Inflammatory Drugs
PCS	Pain Catastrophising Scale
PHQ-9	Patient Health Questionaire-9
SF-36	Short Form 36
TLR4	Toll-Like Receptor 4
TRP	Transient Receptor Potential
TSK	Tampa Scale of Kinesiophobia
